# Fruit and Vegetable Consumption, Fat Intake, and Physical Activity Participation in Relation to Socio-demographic Factors Among Medically Underserved Adults

**DOI:** 10.3934/publichealth.2015.3.402

**Published:** 2015-07-30

**Authors:** Amir A. Hadi Alakaam, Jennifer L. Lemacks

**Affiliations:** 1 Department of Nutrition and Food Systems, The University of Southern Mississippi, Hattiesburg, MS, USA

**Keywords:** fat intake, fruit and vegetable, physical activity, medically underserved, south Mississippi

## Abstract

Fruit and vegetable intake as well as physical activity participation in Mississippi is consistently lower than recommendations. We conducted a cross-sectional study to examine fruit and vegetables consumption, fat intake, and moderate-intensity physical activity participation and how these variables relate to socio-demographic factors among medically underserved adults in south Mississippi. Fruit and vegetable consumption and fat intake along with physical activity participation and socio-demographic characteristics was collected from a sample of 161 (48 male and 113 female) adults in south Mississippi. A majority (81.9%) of participants reported consuming less than five servings of fruits and vegetables per day and 54% reported exercising less than three times a week. Only 14% of participants reported eating a low fat diet. Bivariate correlations revealed no significant relationships between fruit and vegetable consumption and fat intake as well as no significant relationships between fruit and vegetable consumption and gender, ethnicity, income, marital status, or education. However, there were significant correlations between physical activity and fat intake (*r* = -0.21, *p* = 0.01), and physical activity with fruit and vegetable consumption (*r* = 0.16, *p* = 0.05). Higher physical activity rates were associated with decreased fat intake and increased fruit and vegetable consumption. Physical activity was also higher among men (*r* = -0.16, *p* = 0.05*)* and positively correlated with income level (*r* = 0.21 *p* = 0.01). In order to effectively identify or develop strategies to improve health by promoting increased fruit and vegetable intake and physical activity, further research is needed to understand the factors that affect behavior choices regarding nutrition and physical activity in this medically underserved adult population.

## 1. Introduction

According to the Center for Disease Control and Prevention (CDC) [1], approximately 34.6% of the adult population in Mississippi (MS) is considered obese. MS has the lowest rate of Fruit and Vegetable (FV) consumption and Physical Activity (PA) participation compared to other states [2]. Furthermore, several areas within Mississippi are considered as Health Professional Shortage Areas (HPSAs) and vulnerable populations areas [3],[4]. Studies indicate that there are significant increases in the risk of chronic diseases, incidence of cancers, and mortality among medically underserved and ethnic minority populations [3],[5].

Developing health and nutrition programs that target the HPSAs in MS could be helpful in identifying the challenges and barriers to improve the health and nutrition status of this population and may lead to improvement of the overall obesity and chronic disease management in MS. Understanding of dietary intake and physical activity behaviors, as well as the health needs of the vulnerable population from various ethnic groups, is necessary in order to provide effective nutrition and disease management in healthcare [6]. In this study, the researchers examined FV consumption, fat intake, and PA participation among a vulnerable population (the Medically Underserved Adults [MUA] in south Mississippi) as well as described the relationship between these variables. The researchers also described the association between FV consumption, fat intake, and PA participation with socio-demographic factors among this population.

## 2. Materials and Method

The study was approved by the Institutional Review Board for Human Subjects of The University of Southern Mississippi. The study is part of a larger project currently being conducted at the Department of Nutrition and Food Systems that is aiming to improve obesity awareness and management practices in the primary care setting serving rural and economically disadvantaged populations in south Mississippi.

The researchers approached 182 adults at five federally funded healthcare centers (providing general and family practice health care) in south Mississippi with a mission to serve predominantly rural and underserved populations. The researcher collected information from the participants using the droid SURVEY tablet application (a survey software designed for data collection on tablet devices) [7]. The inclusion criteria were: a) at least 21 years of age and b) not pregnant or six months postpartum. The survey took between seven and ten minutes for completion. Full disclosure was provided to participants and none of the participants received any kind of compensation for their participation in the study. The researcher conducted an extensive pilot study to assess the reliability and acceptability of the measures, and modify the questions to elicit more relevant responses.

Initial questions (serving as screening questions) collected information regarding age, gender, height, weight, and gestation status. Based on responses to the initial questions, participants identified as meeting any of the exclusion criteria were immediately redirected to the end of the survey and no data were gathered from these individuals. Eligible participants completed the full survey.

The survey questionnaire included three sections in addition to the screener questions. The first section of the questionnaire measured daily FV consumption, fat intake, and PA participation. The National Cancer Institute (NCI) approach was used to assess the daily intake of FV and fat [8]. This method is specifically developed by NCI to measure dietary intake in a population, and it allows for the combined estimation of both the likelihood and amount of dietary intake in an easy and short format [8]. The approach includes six questions to assess estimated total FV consumption, which asks participants how many 100% juices they drank and number of fruits and vegetables consumed as well as how many servings they usually consumed. Respondents could report their consumption per day, per week, and per month. Responses were rescaled to estimate the frequency of daily fruit and vegetable intake. The NCI method also includes a single question “When you think about the foods you ate over the past 12 months, would you say your diet was high, medium, or low in fat?” to measure fat intake; responses included low, medium, or high.

PA participation was measured with the question: in the past week, how many days have you done a total of 30 minutes or more of physical activity that raised your breathing rate; answers ranged from zero to seven days. This question was developed based on the Dietary Guidelines for Americans [9]; these guidelines recommend that individuals limit leisure time activity and increase physical activity for health benefits. The general recommendation is five or more days per week of moderate-intensity PA at least 30 minutes per day [9].

The second section of the survey measured BMI perception and Stage of Change for weight loss [10]; as well as attitudes, perceived norms, and perceived behavioral control based on the Theory of Planned Behavior principles [11]. The analysis and findings of the second section are presented in another manuscript in preparation [12]. The third section requested demographic information regarding race/ethnicity, marital status, education level, and income level.

Statistical analysis was performed using SPSS (22.0) software. The socio-demographic characteristics of the participants were examined with descriptive statistics. Correlation analyses were used to examine the relationship between FV intake, PA participation, and fat intake. Bivariate correlations were used to assess the relationship between several socio-demographic variables and the FV consumption, fat intakes, and PA participation. The socio-demographic characteristics used in the analysis were: age, gender (female/ male), race (White/ Black/Hispanic), marital status (married/ cohabitating/ divorced/ separated/ single), education levels (“less than high school degree” through “four year college degree or higher”), and income levels ($0–19,999 to $70,000 or above). Statistically significant variables were those with *p*-values less than 0.05, or 95% confidence intervals.

## 3. Results

One hundred and sixty one participants met eligibility criteria (48 men and 113 women). Twenty-one were excluded due to age or pre- or post-partum status. The sample population was quite homogenous, with the majority being female (70.2%), equally Non-Hispanic White (54.7%) and African American (42.2%), and less than 50 years of age (63.9%). Other characteristics of the sample are shown in (Table 1).

**Table 1:** Demographic characteristics of the participants (n = 161).

Characteristic		n	(%)
	21–29	20	12.4
	30–39	52	32.3
	40–49	31	19.3
	50–59	38	23.6
Age	60 or Over	20	12.4
Gender	Male	48	29.8
	Female	113	70.2
Race	White	88	54.7
	Black	68	42.2
	Hispanic or Latino	2	1.2
	I refuse to answer	3	1.9
Marital Status	Married	82	50.9
	Cohabitating	5	3.1
	Divorced	23	14.3
	Separated	7	4.3
	Single	39	24.2
Education Level	Less than high school degree	17	10.9
	A high school degree	52	32.3
	Some college, but not a college degree	40	14.8
	A 2 year or vocational degree	14	8.7
	A 4 year college degree or higher	35	21.7
Income Level	$0 to $19,999	65	40.4
	$20,000 to $29,999	38	23.6
	$30,000 to $39,999	13	8.1
	$40,000 to $49,999	9	5.6
	$50,000 to $59,000	7	4.3
	$60,000 to $69,00	1	0.6
	$70,000 to above	4	2.4
	Currently unemployed	9	5.6

Mean FV intake, fat intake, and PA participation are presented in Table 2. A majority of the participants (81.9%) reported consuming less than five servings of FV per day and only 14% of participants reported eating a low fat diet. Regarding the PA participation, about half of the participants (54%) reported exercising less than three times a week.

**Table 2:** Reported FV intake, Fat intake and PA participation presented as mean.

Variables	N	min	Max	mean	SD
FV intake (servings/day)	160	0	23.25	2.89	3.20
Fat intake^a^	161	1	3	2.04	0.60
PA (times/week ^b^)	161	0	7	2.37	1.98

^a^Fat intake measurement: 1 = low, 2 = medium, 3 = high.

^b^PA refers to how many times participants engaged in at least 30 minutes of moderate intensity PA per week.

Correlations, means, and standard deviations of all variables were calculated to explore associations among different variables. A correlation analysis between PA participation rates with FV and fat intakes, showed a significant positive relationship between PA rate and FV intake (*r* = 0.16, *p* = 0.05); and significant negative relationship between PA rate and fat intake (*r* = -0.21, *p* = 0.01). The correlation analysis showed no significant relationship between FV consumption and fat intake. The correlation between the socio-demographic variables, and the PA participation rates, FV consumption, and fat intakes indicated a significant positive association between PA participation rate and income level (*r* = 0.21 *p* = 0.01). The results also showed that PA participation was significantly higher among men (*r* = -0.16, *p* = 0.05), and there were no significant associations between PA participation rate and ethnicity, education, or marital status. There were also no significant associations between FV and fat intakes with gender, ethnicity, income, marital status, or education. The correlations are shown in Table 3.

**Table 3:** Correlation analysis of FV, Fat intake, PA participation, income, and gender.

	Fat intake	PA	Gender	Income
	*r*-value (*p-*value)n			
FV	0.02 (0.81)160	0.16 (0.05)160	0.04 (0.67)157	0.01 (0.94)136
Fat intake	--	-0.21 (0.01)161	-0.68 (0.39)161	0.01 (0.96)137
PA	--	--	-0.16 (0.04)161	0.22 (0.01)137

*Note*. Statistical significance *p* < 0.05.

## 4. Discussion

The present study examined a MUAs' dietary intake and PA participation rates in cognition with socio-demographic characteristics. Our results indicated that FV intake and PA were relatively low and fat intake was high among underserved adults in south Mississippi. Only twenty percent of the participants reported consuming five servings of FV per day, and only five percent of the participants indicated exercising five times a week. The Dietary Guidelines for American recommends consuming five to six servings per day of fruits and vegetables, as well as 30 minutes of moderate to vigorous activity at least five times a week [9].

It is well documented that consumption of fruits and vegetables and regular physical activity prevent cardiovascular disease, diabetes, several cancers, depression, and obesity [13]–[15]. Furthermore, studies indicated that fruits and vegetables consumption and physical activity are two of the most important factors in disease prevention and health promotion [16],[17]. According to our study, most of our population did not engage in these necessary and essential protective behaviors.

Our results also showed that self-reported PA is higher among men and increased with greater income levels. This finding is similar to other studies conducted [18],[19]; however, these studies showed a positive association between PA and education [18] and negative association between PA and age [19]. Our study did not indicate any significant association among the various levels of education and age with PA. Our sample was fairly distributed among education and age levels across the population. The gender differences in the PA participation can be due to the biological and the psychological variances between men and woman; men have a more positive attitude and interest toward exercise than women, particularly, moderate and vigorous intensity physical activities [17]–[19].

According to our study, higher PA rates were associated with increased FV and decreased fat intakes among all groups, improving modifiable health practices has the potential to lead to improvements in other health practices and could lead to an overall healthy lifestyle among MUA [20]. Previous studies indicated similar associations between individuals' dietary intakes and PA rates where a low intake of FV was associated with low rates of PA participation [20],[21]. This association may be holistic in nature in that active individuals may also have healthy eating behaviors [21] or individuals with sedentary life styles may consume more fast food items and exhibit more unhealthy behaviors [13]. There are many physiological and biological effects related to PA participation, in addition to the appetite regulation, weight control, and reduced risk of cardiovascular diseases; some studies showed that exercise may be a facilitator for other behavior changes [22], such as healthy eating habits and an increase in FV consumption. This influence may be due to the positive regulation of exercise motivation, commitment, efficacy, and confidence among individuals. Some motivational models such as the hierarchical model of motivation (the situational, contextual, and global level) and achievement goal theory have been used in previous studies to explain the influence of exercise on other individual health behaviors [23]. These models suggest that if an individual is self-determined toward PA, this individual will likely be engaged in other behaviors, such as healthy eating habits, which are relevant to his/her health goals [24].

The study suggests that PA participation does not occur in isolation and is correlated with greater FV consumption among this population. Other studies have shown a positive cross-behaviors association between diet intake and PA participation; these studies suggested developing an intervention strategy in any one behavior to facilitate multiple behavior change among individuals [25], [26]. Lippke, Nigg, and Maddock (2012) examined the relationship between nutrition, PA, and smoking behaviors among 3,519 individuals [26]. Findings from this study showed a significant relationship between healthy nutrition behaviors and PA participation. The study concluded that an improvement in one behavior in an individual can help to improve the other behavior [26].

Thus, an intervention could initially target any of the three behaviors (FV consumption, fat intake, or PA) and create a synergetic effect to reduce the risk of chronic disease and obesity among the MUA. Furthermore, our results showed that there were no significant differences in PA rates and fat or FV intake between African Americans or Caucasians who predominantly consisted of individuals with lower income levels. Therefore, improving diet and PA habits in underserved populations may require an intervention specific to individuals with limited resources but can be broad enough to address multiple populations in the same geographic region.

Our study has some limitations. The participants were recruited from a healthcare setting, and some of the participants may suffer from chronic diseases; however, we did not adjust for these conditions since this information was not pertinent to the goals of this study. In addition, the study sample was not a random sample from all the population of south MS, but a real life sample of participants who utilized healthcare services; therefore, the results cannot be generalized to other populations. The relatively low number of men in our population may also be considered another limitation. Our method for exploring physical activity may need to be modified to determine the proportion of other types of physical activity such as aerobic and strength activities [27]. Additionally, a longer procedure might be more valid than the method used in our study to accurately assess individual's fat intake [28] but may be inhibitive in a primary care setting.

This study focused on dietary and physical activity habits of MUAs in south MS. The results indicated that the individuals residing in MUAs have undesirable health habits with low FV consumptions, high fat intake, and low PA participation rate. A promotion of healthier living habits are essential to modify the eating habits and PA behaviors in these individuals. Research among this population should explore the nutrition management services available in the primary care setting serving these individuals and identify the willingness of and availability of resources to primary care providers to address these issues. Strategies may need to be identified to increase provider readiness for lifestyle management in and access to qualified health professionals, such as Registered Dietitians, to provide patient centered care.

## 5. Conclusion

It is important to examine the health and nutrition issues and recognize areas in need of health intervention among the vulnerable community. This study demonstrates that despite efforts to improve lifestyle habits across the U.S., dietary intake and PA participation are still poor among medically underserved communities in south MS. The results suggest that there is a need to improve the health and nutrition services among adults in these areas. An obesity and chronic disease management intervention may be developed to address the needs of underserved populations in south Mississippi despite racial, socioeconomic or gender variability of the population targeted. Further research is needed to identify strategies to improve health habits of underserved populations. Additionally, there is a need to engage underserved males in preventive care and chronic disease management.
